# Ecological impact and metabolic food waste of overweight and obese adults in Northern European and Mediterranean countries

**DOI:** 10.3389/fnut.2025.1505238

**Published:** 2025-04-01

**Authors:** Donato Angelino, Elisabetta Toti, Marina Ramal-Sanchez, Veronica D’Antonio, Chiara Bravo-Trippetta, Mauro Serafini

**Affiliations:** ^1^Department of Bioscience and Technology for Food, Agriculture and Environment, University of Teramo, Teramo, Italy; ^2^Research Center for Food and Nutrition, Council for Agricultural Research and Economics, Rome, Italy

**Keywords:** food sustainability, metabolic food waste, ecological nutrition, obesity, ecological footprints

## Abstract

**Introduction:**

Overeating is one of the main drivers of obesity due to the accumulation of excess body fat (EBF). This issue not only impacts human health but also depletes the planet’s environmental resources through the production of excess food. Thus, the Metabolic Food Waste (MFW) index was developed to measure the food wasted due to EBF accumulation, associated greenhouse gas (GHG) emissions, and the water and land resources used in its production.

**Methods:**

The present study aims to evaluate and compare the MFW and ecological footprints of some Mediterranean countries (MC) and North European countries (NEC). The MFW for six Mediterranean and North European countries (NEC) was calculated using the following: (i) EBF: This is defined as the difference between ideal and actual body weight in overweight and obese adults, with data sourced from the FAOSTAT and WHO databases; (ii) Food waste: This includes the food wasted due to EBF accumulation and energy intake from major food categories. Data for food waste were obtained from the FAO Food Balance Sheets; and (iii) Environmental Impact: This encompasses GHG emissions, water consumption, and land use associated with EBF by different foods. Data were obtained from the WWF Virtual Shopping Cart and the Barilla Centre for Food and Nutrition. Data were analyzed for the total population and standardized per 100,000 citizens.

**Results:**

The results showed that NEC had the highest rates of obesity, while MC recorded slightly higher rates of individuals classified as overweight. Overall, higher EBF values for 100,000 citizens, including both obese and overweight individuals, were found in NEC compared to MC, with smaller population countries predominantly contributing to these trends. Data on MFW and ecological footprints showed that, regardless of the country, the impact of obesity is two to three times greater than that of being overweight. The highest values of MFW and ecological footprints were found in MC, both in the total and among overweight/obese populations.

**Discussion:**

The present study highlights the harmful role of the overeating as on human health as on the resource exploitations of the Earth. In particular, both MC and NEC showed similar alarming data about overeating and, consequently, negative impact on EBF and ecological footprints, suggesting that residence in countries close or far from Mediterranean basin is not a proxy of adherence to healthy dietary patterns. For this reason, informative campaigns should be developed to improve the knowledge on conscious dietary choices for human and planet healthiness.

## 1 Introduction

The World Health Organization (WHO) reported that in 2022, 2.5 billion adults (43% of the global population) were overweight, and among them, 890 million (16% of the global population) were living with obesity (1). Overweight and obesity among adults have also reached epidemic proportions in Europe, where 59% of adults are overweight, with 23% of them classified as obese (1). The WHO defines “overweight” and “obesity” as “*abnormal or excessive fat accumulation that may impair health*” (1). One of the primary causes of this pathological accumulation of body fat is the imbalance between energy intake and expenditure, where excess macronutrients from food are converted into body fat storage. In other words, overconsumption of food directly impacts human health by contributing to body fat accumulation. In 2015, a high body mass index (BMI) contributed to 7% of deaths from all-cause mortality, with cardiovascular diseases, diabetes, and cancer being the main co-morbidities (2), reinforcing the view of overweight and obesity as multifactorial illnesses and risk factors for various other conditions. However, not only is human health affected, but the planet is also affected by the phenomenon of overeating, particularly in terms of the environmental resources wasted in producing excess food. The ecological footprints and resources consumed by each individual’s food and meal choices also affect planetary health. This is particularly relevant when considering food sources, such as plant-based versus animal-based foods, which have different impacts on the health of the planet (3). Additionally, an imbalanced intake of these food types in dietary patterns may negatively affect human health (4).

Robust data from life cycle assessments demonstrate that animal-based products have a greater environmental impact than their plant-based counterparts throughout the entire chain from producer to consumer (3). Considering these aspects, in 2019, the Eat-Lancet Commission developed an example of a “planetary” healthy diet, emphasizing substantial consumption of cereals, fruits, vegetables, legumes, and vegetable oils, along with a reduced intake of animal-based products, such as red and processed meats, which negatively affect both health and the planet (5). However, while numerous markers describe the health and disease status of individuals associated with their overall diet, the health of the planet is still represented by only a few indicators, including the so-called “footprints” (6, 7).

We recently developed a novel index to calculate the ecological impact of overeating – particularly in terms of overweight and obesity conditions, called metabolic food waste (MFW) (8), highlighting the unsustainability of excess body fat. In this study, MFW is identified as the kg of food that corresponds to the quantity leading to excess body fat (EBF) and its environmental impact expressed as carbon [MFW_(kgCO2 eq)_], water [MFW_(L)_], and land footprint [MFW_(m2)_] (8) of 60 overweight and obese Italian individuals. It was then empirically applied to the Italian population with a body mass index (BMI) over 25 kg/m^2^. Another study evaluated MFW in seven FAO regions by comparing the dietary habits of different overweight and obese individuals worldwide and their environmental impacts (9). Our results showed that Europe was responsible for the largest volume of MFW (39.2 million tons), highlighting the importance of understanding the contributions of different EU countries to MFW’s impact on planetary health (9). However, this analysis considered only the entire European continent (and others) rather than individual countries or groups of countries with similar dietary habits, nor did it assess the impact of each overweight or obese individual. Therefore, the present research concentrates on the European peninsula by retrieving data from six Mediterranean countries (MCs), which are expected to follow the sustainable Mediterranean diet, and six Northern European countries (NECs), which do not follow this diet, aiming to assess and compare MFW between the two areas. Moreover, the relative contribution of MFW for the main food commodities will be evaluated while also considering the individual contribution of each overweight or obese adult.

## 2 Materials and methods

The following steps were taken to estimate the MFW for each of the 12 selected countries from two geographic regions: (a) Italy, Spain, France, Portugal, Greece, and Croatia as MC, and (b) Denmark, Iceland, Ireland, Norway, Sweden, and the United Kingdom as NEC.

The most recent data on the total adult population (10), along with the prevalence of overweight and obesity (11) at the national level, has been used to assess the number of individuals classified as overweight or obese based on their BMI categories.

The value of the EBF, expressed in kilograms, was calculated as the difference between the ideal and actual body weight, derived using the BMI inverse function, as described elsewhere (9). Average body weights at the national level were then multiplied by the energy content of 1 kg of body fat (32.2 MJ) to determine the energy from EBF.

Energy from EBF was then associated with kilocalories from food as determined using the Food Balance Sheets (FBS) (10) in FAOSTAT, covering the time period 2010–2019. Food waste was calculated based on the total domestic supply from FBS food items, grouped into nine main groups: dairy products/milk/eggs, starchy roots, alcoholic beverages, cereals, meat/offals, sugar and sweeteners, fish/seafood, added fats, and pulses. Specifically, the percentage energy contribution of each food category was multiplied by the total amount of energy from EBF to determine the energy contribution of each food item contributing to EBF accumulation. This value was then translated into the amount of food wasted due to overweight/obesity, expressed as MFW (_tons of food_).

To determine the total environmental impact of MGW, the following indicators were considered: (a) GHG emissions, measured as carbon footprint (MFW_(kgCO2eq)_), (b) biologically productive land use, measured as land footprint (MFW_(m2 land)_), and (c) water consumption, measured as water footprint (MFW_(L water)_). Data were mainly obtained from the WWF Virtual Shopping Cart (12), with missing data taken from the Barilla Centre for Food and Nutrition (13).

Ethical review and approval were not required for the study involving human participants in accordance with local legislation and institutional guidelines.

Written informed consent from participants was not required to participate in this study in accordance with national legislation and institutional guidelines.

## 3 Results

The descriptive analysis of demographic data, anthropometrics, and EBF for MC and NMC is presented in Table 1. The percentage of overweight citizens in MC is, on average, slightly higher than in NMC, with Italy reporting over 38.4% of the total population in this category. In contrast, NEC exhibited higher obesity rates, with the United Kingdom showing 27.8% of the total population classified as obese.

**Table 1:** Demographic analysis and EBF levels in Mediterranean and Northern European populations.

Country	Total population (*N*)^1^			Overweight and obesity rates (%)^2^		Excess body fat (kg/100,000 citizens)	
	Males	Females	Total	24.9 > BMI > 29.9 kg/m^2^	BMI > 30 kg/m^2^	24.9 > BMI > 29.9 kg/m^2^	BMI > 30 kg/m^2^
Italy	29,456,255	31,206,813	60,663,068	38.4%	19.8%	2.71 × 10^06^	1.02 × 10^07^
Spain	22,887,826	23,746,305	46,634,131	37.7%	23.7%	4.16 × 10^06^	1.05 × 10^07^
France	31,328,637	33,338,953	64,667,590	37.8%	21.5%	3.33 × 10^06^	1.03 × 10^07^
Portugal	4,885,027	5,440,513	10,325,540	36.5%	20.8%	3.20 × 10^06^	9.87 × 10^06^
Greece	5,210,972	5,404,211	10,615,183	37.3%	24.8%	4.55 × 10^06^	1.03 × 10^07^
Croatia	2,024,136	2,184,475	4,208,611	35.0%	24.3%	4.85 × 10^06^	1.01 × 10^07^
Mediterranean countries	**95,792,853**	**101,321,270**	**197,114,123**	**37.8%**	**21.7%**	**2.28 × 10** ^**07**^	**6.12 × 10** ^**07**^
Denmark	2,840,401	2,870,945	5,711,346	35.8%	19.6%	3.53 × 10^06^	1.17 × 10^07^
Iceland	166,644	165,565	332,209	37.2%	21.8%	3.91 × 10^06^	1.14 × 10^07^
Ireland	2,324,524	2,371,266	4,695,790	35.3%	25.3%	5.06 × 10^06^	9.85 × 10^06^
Norway	2,646,258	2,604,692	5,250,950	35.2%	23.1%	4.32 × 10^06^	1.00 × 10^07^
Sweden	4,918,962	4,917,041	9,836,003	35.8%	20.6%	3.50 × 10^06^	1.05 × 10^07^
United Kingdom	32,695,427	33,602,517	66,297,944	35.9%	27.8%	5.68 × 10^06^	9.50 × 10^06^
North Europe countries	**45,592,216**	**46,532,026**	**92,124,242**	**35.7%**	**23.7%**	**2.60 × 10** ^**07**^	**6.31 × 10** ^**07**^

^1^FAO Statistic Division FAOSTAT (10). “Food Balance Sheets” 2019. https://www.fao.org/faostat/en/#data/FBS.

^2^World Health Organization (WHO) (11). “Global Database on Body Mass Index: BMI Classification” 2016.

Bold values, for each column, corresponds to the sum of the values of each single country belonging to Mediterranean or Nothern European countries.

To standardize EBF calculations, data were normalized per 100,000 citizens to account for differences in total population size across countries. In MC, EBF per 100,000 citizens ranged between 2.71 × 10^06^ kg and 4.85 × 10^06^ kg for overweight individuals and 1.02 × 10^07^ kg and 1.05 × 10^07^ kg for obese individuals, with Italy showing the lowest values in both categories. Similarly, in NMC, Denmark had the lowest EBF for overweight individuals and the highest EBF for obese individuals (3.53 × 10^06^ kg and 1.17 × 10^07^ kg, respectively). Then, the United Kingdom had the highest EBF for overweight individuals (5.68 × 10^06^ kg) and the lowest EBF for obese individuals (9.50 × 10^06^ kg) (Table 1).

**Figure 1:**
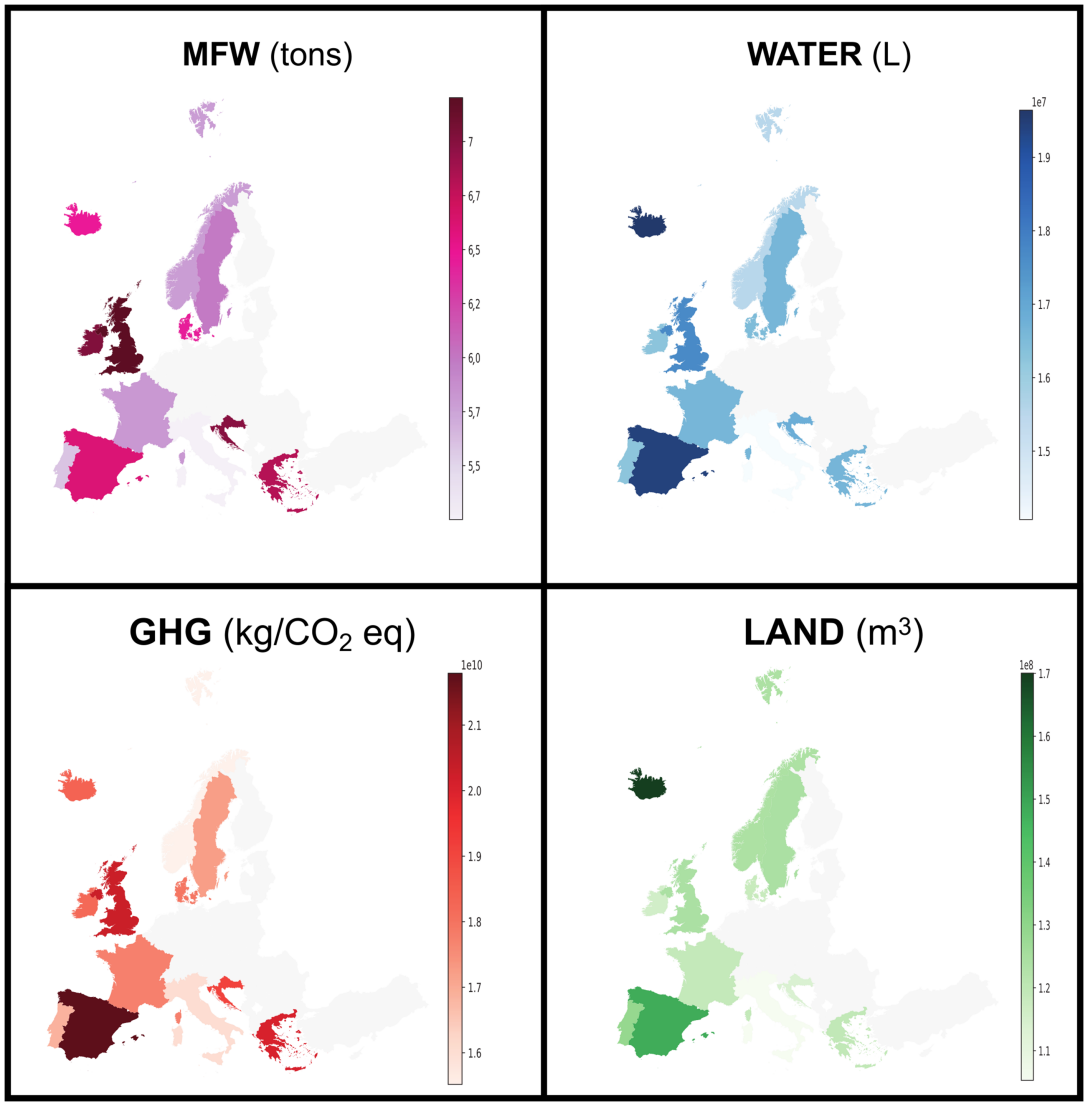
Metabolic food waste expressed as amounts of wasted food (tons), water (L), GHG emissions (kg/CO_2_ eq), and land (m^2^) associated with EBF in the MCs and NCEs. The data are normalized per 100,000 citizens.

In Table 2, the ecological impact of the European countries considered is reported in terms of MFW (kg of food) as well as CO_2_, water, and land footprints. Overall, the values of MFW and all the other parameters of the ecological impact of MC are twofold higher than those of NEC in the total population. Among the countries, France, Spain, and Italy were responsible for the highest values of MFW (>3.0 × 10^03^ tons), water (~1.0 × 10^13^ L), and CO_2_ (~1.0 × 10^10^ kg/CO_2_ eq) in MC. In contrast, the UK showed the highest values of MFW (4.9 × 10^03^ tons), water (1.4 × 10^13^ L), CO_2_ (1.2 × 10^10^ kg/CO_2_ eq), and land (7.4 × 10^10^ m^2^) among NC, followed by the Scandinavian countries. However, the countries show wide differences in total population, and normalizing the data per citizen may be appropriate. In Figure 1, the ecological impact of the European countries considered is represented geographically in terms of metabolic food waste, as well as CO_2_, water, and land footprints per 100,000 citizens. The results were quite similar when comparing MC and NEC. Specifically, MFW was 3.71 × 10^1^ *vs.* 3.89 × 10^1^ tons of food waste, respectively. However, it emerged that even small countries are significant contributors to the ecological impact of EBF. In fact, not only Spain but also Croatia and Greece exhibited the highest amounts of MFW (7.00 and 6.82 tons of food waste) and CO_2_ (2.01 × 10^10^ and 1.91 × 10^10^ kg CO_2_ eq) and water (1.69 × 10^7^ and 1.67 × 10^7^ L) impacts. Similarly, among the NEC, apart from the UK, Ireland, and Iceland displayed the highest amounts of MFW (7.03 and 6.47 tons of food waste) and CO_2_ (1.83 × 10^10^ and 1.84 × 10^10^ kg CO_2_ eq) impacts.

**Table 2:** Metabolic food waste expressed as amounts of wasted food (tons) and GHG emissions (kg CO_2_eq), as well as water (L) and land (m^2^) corresponding to EBF in Mediterranean and Northern European countries.

	Total				Overweight (24.9 < BMI < 30 kg/m^2^)				Obese (BMI > 30 kg/m^2^)			
Country	MFW (tons)	CO_2_ (kg)	Water (L)	ECO (m^2^)	MFW (tons)	CO_2_ (kg)	Water (L)	ECO (m^2^)	MFW (tons)	GHG (kg)	Water (L)	ECO (m^2^)
Italy	3.2 × 10^03^	8.5 × 10^09^	9.7 × 10^12^	5.3 × 10^10^	6.3 × 10^02^	1.7 × 10^09^	1.9 × 10^12^	1.1 × 10^10^	1.2 × 10^03^	3.3 × 10^09^	3.7 × 10^12^	2.0 × 10^10^
Spain	3.1 × 10^03^	9.1 × 10^09^	1.0 × 10^13^	5.5 × 10^10^	7.3 × 10^02^	2.1 × 10^09^	2.4 × 10^12^	1.3 × 10^10^	1.2 × 10^03^	3.4 × 10^09^	3.8 × 10^12^	2.1 × 10^10^
France	3.8 × 10^03^	1.1 × 10^10^	1.2 × 10^13^	6.4 × 10^10^	8.1 × 10^02^	2.3 × 10^09^	2.5 × 10^12^	1.4 × 10^10^	1.4 × 10^03^	4.1 × 10^09^	4.4 × 10^12^	2.4 × 10^10^
Portugal	5.8 × 10^02^	1.7 × 10^09^	1.7 × 10^12^	9.7 × 10^09^	1.2 × 10^02^	3.5 × 10^08^	3.6 × 10^11^	2.0 × 10^09^	2.1 × 10^02^	6.1 × 10^08^	6.4 × 10^11^	3.5 × 10^09^
Greece	7.3 × 10^02^	1.8 × 10^09^	2.1 × 10^12^	1.1 × 10^10^	1.8 × 10^02^	4.4 × 10^08^	5.3 × 10^11^	2.8 × 10^09^	2.7 × 10^02^	6.6 × 10^08^	8.0 × 10^11^	4.2 × 10^09^
Croatia	2.9 × 10^02^	7.1 × 10^08^	8.0 × 10^11^	4.2 × 10^09^	7.2 × 10^01^	1.7 × 10^08^	2.0 × 10^11^	1.0 × 10^09^	1.0 × 10^02^	2.5 × 10^08^	2.8 × 10^11^	1.5 × 10^09^
Mediterranean countries	**1.2 × 10** ^**7**^	**3.3 × 10** ^**10**^	**3.6 × 10** ^**13**^	**2.0 × 10** ^**11**^	**2.5 × 10** ^**03**^	**7.1 × 10** ^**09**^	**7.9 × 10** ^**12**^	**4.3 × 10** ^**10**^	**4.4 × 10** ^**03**^	**1.2 × 10** ^**10**^	**1.4 × 10** ^**13**^	**7.4 × 10** ^**10**^
Denmark	3.7 × 10^02^	9.9 × 10^08^	1.0 × 10^12^	5.8 × 10^09^	7.2 × 10^01^	1.9 × 10^08^	2.0 × 10^11^	1.1 × 10^09^	1.3 × 10^02^	3.6 × 10^08^	3.7 × 10^11^	2.1 × 10^09^
Iceland	2.2 × 10^1^	6.5 × 10^07^	6.2 × 10^10^	3.7 × 10^08^	4.8 × 10^01^	1.4 × 10^07^	1.4 × 10^10^	8.0 × 10^07^	8.2	2.4 × 10^07^	2.3 × 10^10^	1.4 × 10^08^
Ireland	3.3 × 10^02^	7.7 × 10^08^	8.6 × 10^12^	4.8 × 10^09^	8.4 × 10^01^	1.9 × 10^08^	2.2 × 10^11^	1.2 × 10^09^	1.2 × 10^02^	2.7 × 10^08^	3.0 × 10^11^	1.7 × 10^09^
Norway	3.5 × 10^02^	8.2 × 10^08^	8.9 × 10^12^	5.3 × 10^09^	8.0 × 10^01^	1.9 × 10^08^	2.1 × 10^11^	1.2 × 10^09^	1.2 × 10^02^	2.9 × 10^08^	3.1 × 10^11^	1.9 × 10^09^
Sweden	6.0 × 10^02^	1.6 × 10^09^	1.7 × 10^12^	1.0 × 10^10^	1.2 × 10^02^	3.4 × 10^08^	3.5 × 10^11^	2.1 × 10^09^	2.1 × 10^02^	5.9 × 10^08^	6.1 × 10^11^	3.6 × 10^09^
UK	4.9 × 10^03^	1.2 × 10^10^	1.4 × 10^13^	7.4 × 10^10^	1.4 × 10^03^	3.3 × 10^09^	3.8 × 10^12^	2.1 × 10^10^	1.7 × 10^03^	4.2 × 10^09^	4.9 × 10^12^	2.7 × 10^10^
Northern European countries	**6.5 × 10** ^**06**^	**1.6 × 10** ^**10**^	**1.8 × 10** ^**13**^	**1.0 × 10** ^**11**^	**1.7 × 10** ^**03**^	**4.2 × 10** ^**09**^	**4.8 × 10** ^**12**^	**2.6 × 10** ^**10**^	**2.3 × 10** ^**03**^	**5.7 × 10** ^**09**^	**6.5 × 10** ^**12**^	**3.6 × 10** ^**10**^

Data are shown for the total population, including overweight and obese individuals. BMI (Body Mass Index); MFW (Metabolic Food Waste, expressed in kg of food per x kg of EBF); Water (Water Footprint, expressed in liters); CO^2^ (GHG emissions, expressed in kg of CO_2_); ECO (Ecological Footprint, expressed in m^2^).

Bold values, for each column, corresponds to the sum of the values of each single country belonging to Mediterranean or Nothern European countries.

**Figure 2:**
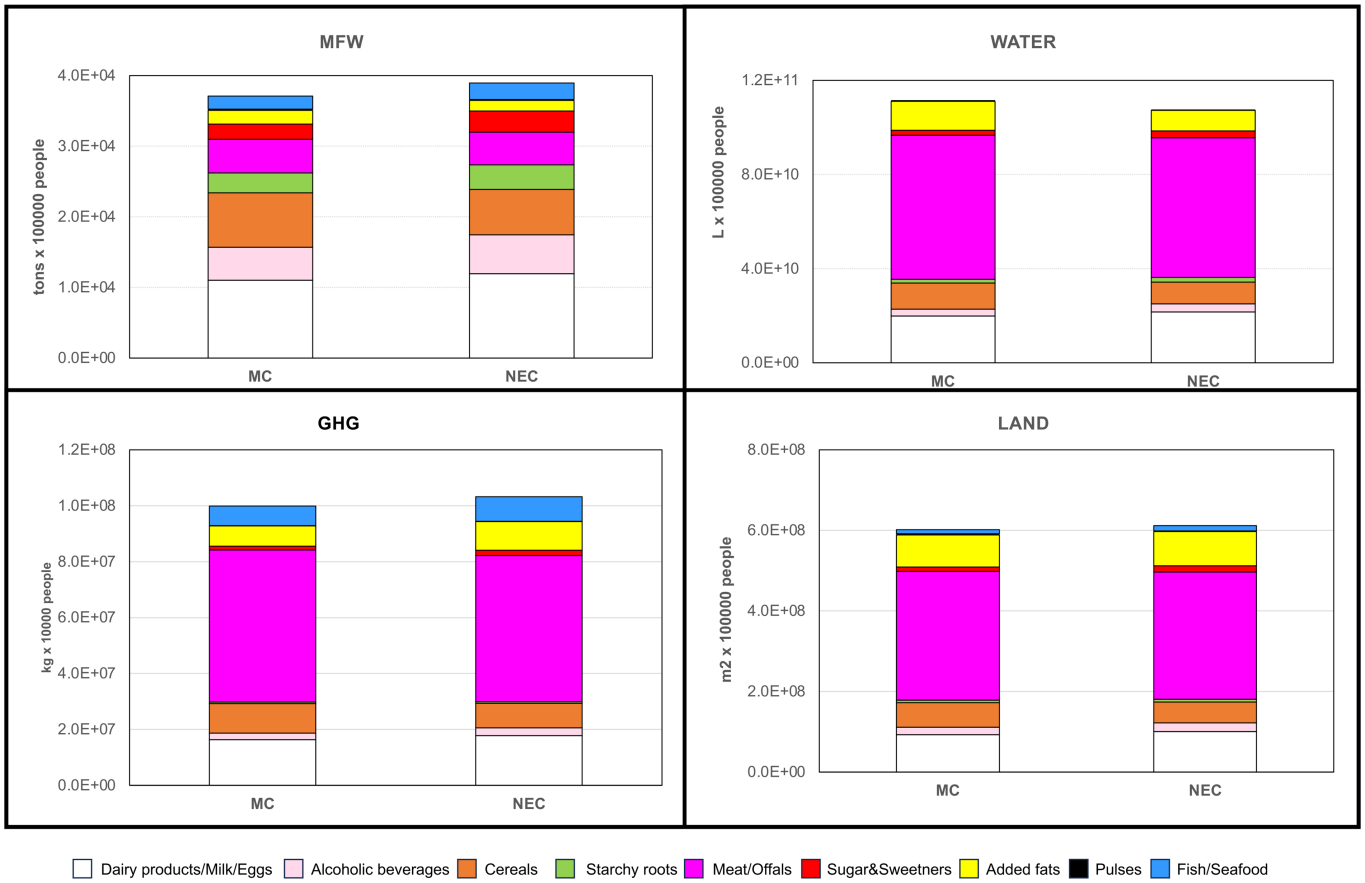
Metabolic Food waste expressed as amounts of wasted food (kg) and as GHG emissions (kg CO2 eq), water consumption (L), and land use (m2) for the overweight and obese populations in the Mediterranean and Northern European regions.

When considering only the whole overweight and obese populations, data confirmed a greater impact of total MCs compared to NECs, with nearly double values for obese individuals (Table 1). The countries most affected by MCs are identified as France, Italy, and Spain, while no significant differences are evident among NECs. To standardize the data for individual cases in each country, we presented the MFW (expressed in kg instead of tons for increased clarity) and the ecological footprints per overweight and obese individual for each country considered in Table 3. Overall, average values indicate a greater impact of MCs compared to NECs. However, similar results were found when comparing MCs and NEC among obese individuals (Table 3). Within MCs, overweight individuals showed some variabilities depending on the country: Greek and Croatian overweight individuals exhibit nearly double MFW values and ecological footprints compared to their Italian counterparts. Similarly, within NECs, Ireland and the UK show higher MFW and ecological footprint values than other countries. No substantial differences are noted within the northern and MC categories (Table 3). Figure 2 summarizes the MFW and ecological impact data by considering all MCs and NECs overweight and obese individuals. Overall, negligible differences are found between MCs and NECs overweight individual values, as well as between MCs and NECs obese values. However, when comparing values for overweight and obese individuals—regardless of MCs or NECs—the impact values for obese individuals are two to three times higher than those for overweight individuals (Figure 2).

**Table 3:** Metabolic food waste expressed as amounts of wasted food (kg) and GHG emissions (kg CO_2_ eq), water (L), and land (m^2^) associated with EBF for each overweight and obese individual living in MCs and NECs.

	Overweight (24.9 < BMI < 30 kg/m^2^)				Obese (BMI > 30 kg/m^2^)			
Country	MFW (kg)	CO_2_ (kg)	Water (L)	ECO (m^2^)	MFW (kg)	GHG (kg)	Water (L)	ECO (m^2^)
Mediterranean countries								
Italy	27.1	72.5	8.3 × 10^04^	451	102	273	3.1 × 10^05^	1697
Spain	41.6	122	1.4 × 10^05^	742	105	309	3.5 × 10^05^	1878
France	33.3	94.8	1.0 × 10^05^	560	103	292	3.1 × 10^05^	1726
Portugal	32.0	92.8	9.6 × 10^04^	534	98.7	286	3.0 × 10^05^	1646
Greece	45.5	111	1.3 × 10^05^	698	103	251	3.0 × 10^05^	1577
Croatia	48.5	117	1.3 × 10^05^	699	101	243	2.8 × 10^05^	1451
Average values (Mean ± SD)	**38.0 ± 8.45**	**102 ± 18.5**	**1.1 × 10**^**5**^ **± 2.3 10**^**4**^	**613 ± 115**	**102 ± 2.21**	**276 ± 25.5**	**3.1 × 10**^**5**^ **± 2.4 10**^**4**^	**1663 ± 144**
Northern European countries								
Denmark	35.3	95.2	9.9 × 10^04^	559	118	317	3.3 × 10^05^	1861
Iceland	39.1	115	1.1 × 10^05^	649	114	335	3.2 × 10^05^	1889
Ireland	50.6	117	1.3 × 10^05^	736	99	227	2.6 × 10^05^	1432
Norway	43.2	102	1.1 × 10^05^	664	101	238	2.6 × 10^05^	1546
Sweden	35.0	96.0	1.0 × 10^05^	595	106	289	3.0 × 10^05^	1795
UK	56.8	137	1.6 × 10^05^	863	95.0	229	2.7 × 10^05^	1442
Average values (Mean ± SD)	**43.3 ± 8.81**	**110 ± 16.0**	**1.2 × 10**^**5**^ **± 2.3 10**^**4**^	**678 ± 109**	**105 ± 8.91**	**273 ± 47.5**	**2.9 × 10**^**5**^ **± 3.2 10**^**4**^	**1661 ± 212**

Bold values, for each column, corresponds to the sum of the values of each single country belonging to Mediterranean or Nothern European countries.

**Figure 3:**
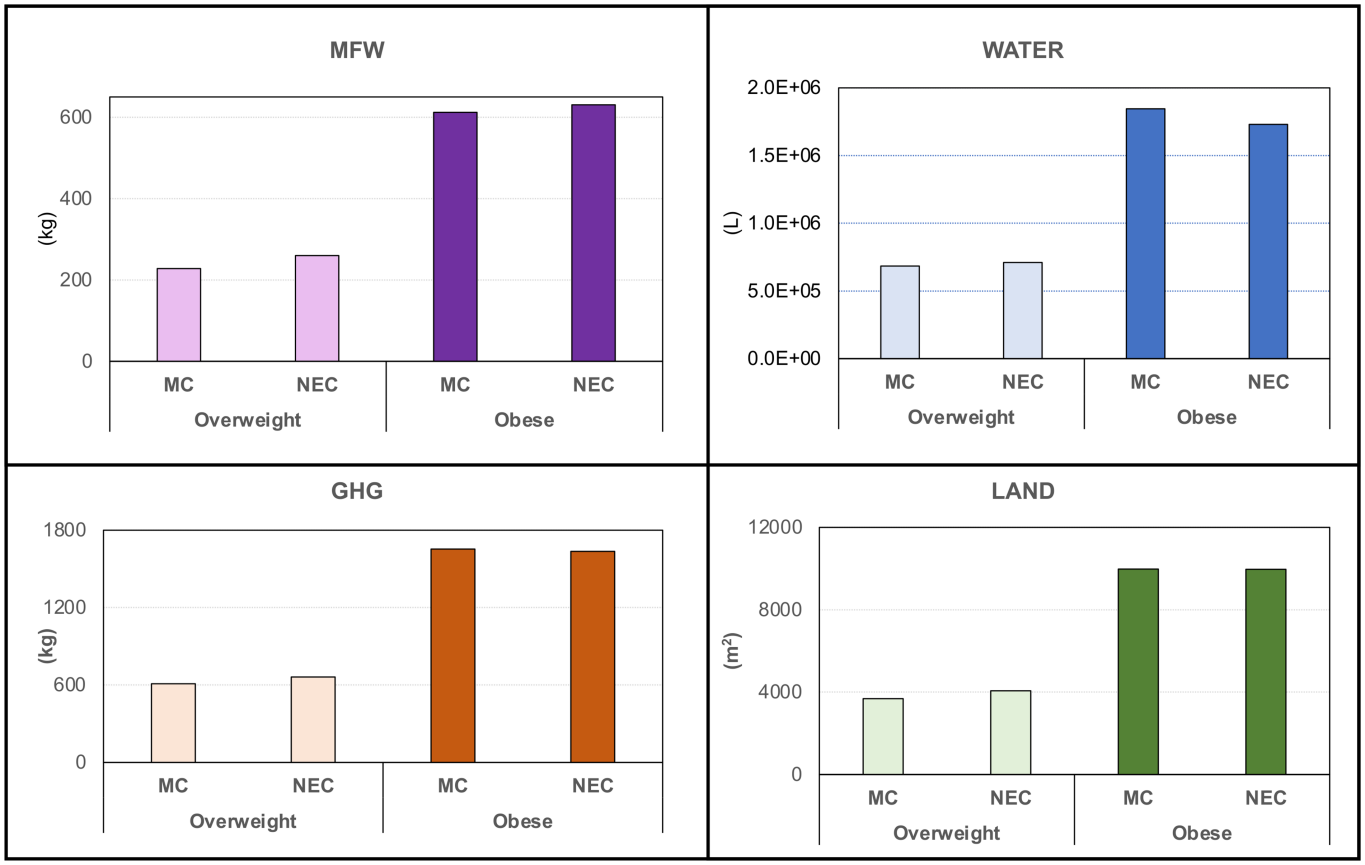
MFW corresponds to EBF from food balance sheet commodities in a population of 100,000 overweight and obese individuals living in Mediterranean (MC) or North European (NEC) countries. The data are expressed as tons per 100,000 citizens of wasted foods (MFW), liters per 100,000 citizens of water (WATER), kgCO_2_eq per 100,000 citizens (GHG), and millions of m_2_ per 100,000 citizens (LAND).

The analysis of the food groups contributing to the EBF of 100,000 citizens of the considered countries is shown in Figure 3. Dairy products/milk/eggs had a high impact on food waste, with MC countries accounting for ~1.1 × 10^4^ tons of wasted food (29.7% of the total) and NEC countries accounting for ~1.2 × 10^4^ tons of food wasted (30.7% of the total).

Alcoholic beverages, cereals, and meat/offals were also among the most impactful food groups, with a combined waste of ~1.7 10^3^ tons (46.1% of the total) in MC and ~1.6 10^3^ tons (42.4% of the total) in NEC. Conversely, meat/offals were the largest contributors, accounting for ~50 to 55% of the total ecological impact due to MFW, with very similar values as in MCs and NECs, water footprint: ~6 × 10^10^ L, carbon footprint: ~5 × 10^7^ kg/CO_2_ eq, and land footprint: ~3 × 10^8^ m^2^ (Figure 3). In particular, Spain and France were the main contributors to the MFW ecological impact from animal-based food groups, while the UK and Iceland had the greatest impact due to high meat consumption and its derivatives (Supplementary Table 1).

## 4 Discussion

The main finding of this study supports the role of malnutrition in affecting not only human health but also the planet’s health, confirming the intricate and inseparable link between human dietary habits and the planet’s response to overeating. Demographic data indicate that more than half of European citizens are in a state of overnutrition. Although MCs have double the number of citizens compared to NECs, data show similar percentages of overweight and obese individuals in both groups, with peaks of 38.8 and 27.8%, respectively. Additionally, the two conditions do not impact the planet’s environmental resources similarly, as obese individuals have EBF and MFW values at least three times higher than those of overweight individuals. This aspect has also been confirmed by data from the NHANES cohort, where the “sustainable diet index-US” was inversely associated with higher odds of obesity (14). Normalizing MFW and ecological footprints per 100,000 citizens allowed us to avoid bias due to differences in the populations of the countries, leading to two main results: (i) small countries are significant contributors to MFW and emissions and (ii) there is not a substantial difference in the sustainability of the diets of overweight and obese individuals living in MCs and NECs. This latter aspect is worth discussing, as historically, countries in the Mediterranean basin were characterized by strict adherence to the Mediterranean diet, one of the most sustainable dietary patterns for human and planetary health (15). However, in recent decades, research has shown that adherence to the Mediterranean Diet among citizens living in MCs has significantly decreased, particularly in countries such as Spain, Italy, Greece, and France. Conversely, NEC countries such as Denmark, Norway, and even the UK have seen a slight increase in their adherence to this diet (16). Castaldi et al. (17) clearly described a significant deviation of some MCs from the ideal Medi*terranean diet in terms of fo*od intake and GHG emissions. This was primarily due to higher intakes of cereals, added fats, and red and poultry meats compared to the ideal Mediterranean diet, demonstrating a three-fold increase in GHG emissions from 1960 to 2010 attributable solely to the rise in meat consumption (mainly pork and poultry), together with other 21 factors (17). Such data have also been corroborated by the present study, where meat/offals and added fats are primarily responsible for carbon, water, and land waste in both MCs and NECs.

Except for low consumption of animal-based products, the Mediterranean diet is globally recognized for its rich daily intake of vegetables and minimally processed foods, which play a dual role in enhancing human health and reducing environmental footprints compared to other dietary patterns (18). Although the primary vegetable-based food categories are not included in this study—as they do not significantly contribute to EBF in individuals—the consumption of fruits, vegetables, cereals, and nuts is declining in MCs, posing a risk factor for obesity and other chronic diseases. Surveys conducted using the MEDAS questionnaire indicated that MC and NECs do not differ significantly in their low intake of fruits (< 3 servings/day), vegetables (< 2 servings/day), and nuts (< 3 servings/week) (19). This study also suggests a need to decrease the consumption of animal-based products and reverse the declining trend in vegetable intake to lower EBF and MFW overall. A French study also aimed to quantify the total food amounts and individual food categories that should be adjusted in certain MC and NEC to achieve ecological adequacy for GHG emissions (20). Overall, at least a 1 kg/day change in total absolute food weight was required to reach a nutritionally adequate and sustainable diet, with only minor differences between MC and NEC. Regarding food categories, an increased energy intake and GHG emissions from fruits and vegetables, alongside a decreased energy intake and GHG emissions from the sugar/fat/alcohol food group, are necessary to achieve this goal (20).

While MCs are experiencing a decline in adherence to the Mediterranean diet, there is a growing interest in NECs adopting the Nordic Diet (21). Due to the high intake of animal-based products in NECs, this relatively new sustainable dietary pattern promotes a shift toward increased consumption of plant-based foods over animal-based ones, particularly focusing on locally sourced vegetables, such as berries, cabbages, root vegetables, and legumes while also ensuring an adequate intake of fish as a source of protein and unsaturated fats (21). Adhering to this dietary pattern may be—and should be—an effective strategy to reduce malnutrition and lower the ecological impact of diets in these countries (22, 23). Moreover, the authors suggest that it can also be considered a healthy and sustainable option in southern European countries (24).

The present study shows some methodological limitations. First, by using epidemiological data from WHO databases on the anthropometrics of European countries, the level of EBF has been empirically calculated by assuming that the difference between the ideal and actual BMI of citizens is theoretically represented by fat mass. This assumption may not be accurate, as a single value of body weight—and consequently BMI—does not clearly indicate which tissue constitutes the weight. Another limitation is the heterogeneity among data sources and the availability of different databases evaluating the ecological footprints of foods, which may affect the actual calculation of the environmental impact of food and food overconsumption (25). Finally, data regarding the intake of food groups may be biased since food balance sheets report the availability of food commodities rather than consumption or intake data.

## 5 Conclusion

The present study confirms the utility and reliability of the MFW as a tool for describing the contribution of overeating not only to promoting overweight and obesity but also to quantifying the amount of MFW in terms of GHG emissions, as well as water and land consumption resulting from excess food production. In the future, this tool may be integrated into the daily practices of healthcare professionals to evaluate not only the healthiness of subjects’ diets but also their environmental sustainability.

Data indicate a shift in the dietary habits of European citizens, particularly showing that MC countries seem to be less sustainable than NEC countries. This situation arises from high rates of overweight and obese individuals, coupled with the overconsumption of food categories that are not typical of the Mediterranean diet, recognized as one of the most sustainable dietary patterns. Such conclusions should encourage the planning of public information campaigns and initiatives—targeting both children and adults—to enhance the understanding of the role of lifestyle, particularly diet, in the crucial link between human health and the planet’s health. Citizens should remain aware that their dietary choices and caloric intake from unbalanced diets increase the risk of obesity and related metabolic diseases and contribute to unnecessary ecological footprints that affect the planet’s health.

## Data Availability

The original contributions presented in the study are included in the article/Supplementary material, further inquiries can be directed to the corresponding author.
