# Supplementary material for: Ecological impact and metabolic food waste of overweight and obese adults in Northern European and Mediterranean countries
Source: Front Nutr. 2025 Apr 1;12:1505238. doi: 10.3389/fnut.2025.1505238 (PMC11996667; doi:10.3389/fnut.2025.1505238)
Supplement: Supplementary file 1 [file Table_1.DOCX]

**Suppl Table 1.** **Metabolic Food Waste expressed as amounts of different food/food categories wasted (tons) and as GHG emissions (kg/CO2 eq), water (L) and land (m^2^) corresponding to Excess Body Fat in Mediterranean and Northern European Countries.** Data are normalized per 100.000 citizens

| **Country** | **Item** | **MFW (Tons)** | **GHG Footprint (kg)** | **Water Footprint (L)** | **Ecological Footprint (m^2^)** |
| --- | --- | --- | --- | --- | --- |
| Italy | Starchy Roots | 294 | 5.4E+04 | 1.6E+08 | 5.9E+05 |
|  | Offals | 19 | 3.6E+05 | 2.9E+08 | 3.5E+05 |
|  | Vegetable Oils | 225 | 6.3E+05 | 1.7E+09 | 9.7E+06 |
|  | Pulses | 43 | 4.0E+04 | 1.2E+08 | 6.5E+05 |
|  | Meat | 621 | 6.9E+06 | 7.9E+09 | 4.3E+07 |
|  | Milk-Excluding Butter | 1497 | 2.1E+06 | 2.6E+09 | 1.2E+07 |
|  | Sugar&Sweeteners | 270 | 1.7E+05 | 2.6E+08 | 1.4E+06 |
|  | Alcoholic Beverages | 501 | 2.5E+05 | 3.2E+08 | 2.0E+06 |
|  | Eggs | 97 | 3.1E+05 | 3.2E+08 | 1.5E+06 |
|  | Animal Fats | 44 | 4.6E+05 | 2.3E+08 | 3.3E+06 |
|  | Cereals-Excluding Beer | 1356 | 1.9E+06 | 2.0E+09 | 1.1E+07 |
|  | Fish, Seafood | 248 | 9.5E+05 |  | 1.4E+06 |
|  | Miscellaneous | 6 | 3.0E+02 | 1.1E+07 | 9.7E+04 |
|  | Oilcrops | 35 | 2.1E+04 | 1.3E+08 | 3.2E+05 |
|  | **Total** | **5257** | **1.4E+07** | **1.6E+10** | **8.7E+07** |
| Spain | Starchy Roots | 579 | 1.1E+05 | 3.2E+08 | 1.2E+06 |
|  | Offals | 38 | 7.4E+05 | 5.8E+08 | 7.2E+05 |
|  | Vegetable Oils | 281 | 7.9E+05 | 2.2E+09 | 1.2E+07 |
|  | Pulses | 50 | 4.7E+04 | 1.4E+08 | 7.5E+05 |
|  | Meat | 968 | 1.1E+07 | 1.2E+10 | 6.8E+07 |
|  | Milk-Excluding Butter | 1589 | 2.2E+06 | 2.7E+09 | 1.3E+07 |
|  | Sugar&Sweeteners | 332 | 2.1E+05 | 3.2E+08 | 1.7E+06 |
|  | Alcoholic Beverages | 986 | 4.9E+05 | 6.3E+08 | 3.9E+06 |
|  | Eggs | 146 | 4.6E+05 | 4.7E+08 | 2.2E+06 |
|  | Animal Fats | 43 | 4.5E+05 | 2.3E+08 | 3.2E+06 |
|  | Cereals-Excluding Beer | 1129 | 1.5E+06 | 1.6E+09 | 9.0E+06 |
|  | Fish, Seafood | 421 | 1.6E+06 |  | 2.4E+06 |
|  | Miscellaneous |  |  |  |  |
|  | Oilcrops | 50 | 3.0E+04 | 1.9E+08 | 4.5E+05 |
|  | **Total** | **6612** | **1.9E+07** | **2.2E+10** | **1.2E+08** |
| France | Starchy Roots | 444 | 8.2E+04 | 2.5E+08 | 8.9E+05 |
|  | Offals | 54 | 1.1E+06 | 8.3E+08 | 1.0E+06 |
|  | Vegetable Oils | 151 | 4.3E+05 | 1.2E+09 | 6.5E+06 |
|  | Pulses | 14 | 1.3E+04 | 3.7E+07 | 2.0E+05 |
|  | Meat | 710 | 7.9E+06 | 9.1E+09 | 5.0E+07 |
|  | Milk-Excluding Butter | 1567 | 2.2E+06 | 2.7E+09 | 1.3E+07 |
|  | Sugar&Sweeteners | 345 | 2.1E+05 | 3.3E+08 | 1.7E+06 |
|  | Alcoholic Beverages | 678 | 3.4E+05 | 4.3E+08 | 2.7E+06 |
|  | Eggs | 100 | 3.2E+05 | 3.2E+08 | 1.5E+06 |
|  | Animal Fats | 118 | 1.2E+06 | 6.2E+08 | 8.7E+06 |
|  | Cereals-Excluding Beer | 1327 | 1.8E+06 | 1.9E+09 | 1.1E+07 |
|  | Fish, Seafood | 291 | 1.1E+06 |  | 1.6E+06 |
|  | Miscellaneous | 27 | 1.3E+03 | 4.9E+07 | 4.3E+05 |
|  | Oilcrops | 23 | 1.4E+04 | 8.7E+07 | 2.1E+05 |
|  | **Total** | **5845** | **1.7E+07** | **1.8E+10** | **9.8E+07** |
| Portugal | Starchy Roots | 555 | 1.0E+05 | 3.1E+08 | 1.1E+06 |
|  | Offals | 37 | 7.3E+05 | 5.8E+08 | 7.1E+05 |
|  | Vegetable Oils | 128 | 3.6E+05 | 9.9E+08 | 5.5E+06 |
|  | Pulses | 30 | 2.8E+04 | 8.1E+07 | 4.5E+05 |
|  | Meat | 767 | 8.5E+06 | 9.8E+09 | 5.4E+07 |
|  | Milk-Excluding Butter | 1144 | 1.6E+06 | 2.0E+09 | 9.2E+06 |
|  | Sugar&Sweeteners | 345 | 2.1E+05 | 3.3E+08 | 1.7E+06 |
|  | Alcoholic Beverages | 842 | 4.2E+05 | 5.4E+08 | 3.4E+06 |
|  | Eggs | 77 | 2.5E+05 | 2.5E+08 | 1.2E+06 |
|  | Animal Fats | 65 | 6.8E+05 | 3.4E+08 | 4.8E+06 |
|  | Cereals-Excluding Beer | 1099 | 1.5E+06 | 1.6E+09 | 8.8E+06 |
|  | Fish, Seafood | 491 | 1.9E+06 |  | 2.8E+06 |
|  | Miscellaneous | 17 | 8.6E+02 | 3.1E+07 | 2.7E+05 |
|  | Oilcrops | 17 | 1.0E+04 | 6.1E+07 | 1.5E+05 |
|  | **Total** | **5616** | **1.6E+07** | **1.7E+10** | **9.4E+07** |
| Greece | Starchy Roots | 568 | 1.0E+05 | 3.1E+08 | 1.1E+06 |
|  | Offals | 37 | 7.2E+05 | 5.6E+08 | 7.0E+05 |
|  | Vegetable Oils | 286 | 8.0E+05 | 2.2E+09 | 1.2E+07 |
|  | Pulses | 50 | 4.7E+04 | 1.4E+08 | 7.6E+05 |
|  | Meat | 697 | 7.7E+06 | 8.9E+09 | 4.9E+07 |
|  | Milk-Excluding Butter | 2342 | 3.2E+06 | 4.0E+09 | 1.9E+07 |
|  | Sugar&Sweeteners | 300 | 1.9E+05 | 2.9E+08 | 1.5E+06 |
|  | Alcoholic Beverages | 586 | 2.9E+05 | 3.7E+08 | 2.3E+06 |
|  | Eggs | 91 | 2.9E+05 | 3.0E+08 | 1.4E+06 |
|  | Animal Fats | 36 | 3.7E+05 | 1.9E+08 | 2.6E+06 |
|  | Cereals-Excluding Beer | 1420 | 1.9E+06 | 2.1E+09 | 1.1E+07 |
|  | Fish, Seafood | 211 | 8.1E+05 |  | 1.2E+06 |
|  | Miscellaneous | 23 | 1.1E+03 | 4.1E+07 | 3.6E+05 |
|  | Oilcrops | 194 | 1.2E+05 | 7.2E+08 | 1.7E+06 |
|  | **Total** | **6840** | **1.7E+07** | **2.0E+10** | **1.0E+08** |
| Croatia | Starchy Roots | 417 | 7.7E+04 | 2.3E+08 | 8.3E+05 |
|  | Offals | 40 | 7.8E+05 | 6.1E+08 | 7.6E+05 |
|  | Vegetable Oils | 93 | 2.6E+05 | 7.2E+08 | 4.0E+06 |
|  | Pulses | 8 | 7.2E+03 | 2.1E+07 | 1.2E+05 |
|  | Meat | 747 | 8.3E+06 | 9.6E+09 | 5.2E+07 |
|  | Milk-Excluding Butter | 2272 | 3.1E+06 | 3.9E+09 | 1.8E+07 |
|  | Sugar&Sweeteners | 575 | 3.6E+05 | 5.6E+08 | 2.9E+06 |
|  | Alcoholic Beverages | 1069 | 5.3E+05 | 6.8E+08 | 4.3E+06 |
|  | Eggs | 103 | 3.3E+05 | 3.4E+08 | 1.5E+06 |
|  | Animal Fats | 44 | 4.5E+05 | 2.3E+08 | 3.2E+06 |
|  | Cereals-Excluding Beer | 1371 | 1.9E+06 | 2.0E+09 | 1.1E+07 |
|  | Fish, Seafood | 182 | 6.9E+05 |  | 1.0E+06 |
|  | Miscellaneous | 0 |  |  |  |
|  | Oilcrops | 76 | 4.6E+04 | 2.8E+08 | 6.9E+05 |
|  | **Total** | **7.0E+03** | **1.7E+07** | **1.9E+10** | **1.0E+08** |
| **Total Mediterranean Countries** | Starchy Roots | 2856 | 5.3E+05 | 1.6E+09 | 5.7E+06 |
|  | Offals | 224 | 4.4E+06 | 3.4E+09 | 4.3E+06 |
|  | Vegetable Oils | 1164 | 3.3E+06 | 9.0E+09 | 5.0E+07 |
|  | Pulses | 196 | 1.8E+05 | 5.3E+08 | 2.9E+06 |
|  | Meat | 4510 | 5.0E+07 | 5.8E+10 | 3.2E+08 |
|  | Milk-Excluding Butter | 10411 | 1.4E+07 | 1.8E+10 | 8.3E+07 |
|  | Sugar&Sweeteners | 2167 | 1.3E+06 | 2.1E+09 | 1.1E+07 |
|  | Alcoholic Beverages | 4663 | 2.3E+06 | 3.0E+09 | 1.9E+07 |
|  | Eggs | 614 | 2.0E+06 | 2.0E+09 | 9.2E+06 |
|  | Animal Fats | 350 | 3.6E+06 | 1.8E+09 | 2.6E+07 |
|  | Cereals-Excluding Beer | 7701 | 1.1E+07 | 1.1E+10 | 6.2E+07 |
|  | Fish, Seafood | 1844 | 7.0E+06 |  | 1.0E+07 |
|  | Miscellaneous | 72 | 3.6E+03 | 1.3E+08 | 1.2E+06 |
|  | Oilcrops | 396 | 2.4E+05 | 1.5E+09 | 3.6E+06 |
|  | **TOTAL** | **3.7E+04** | **1.0E+08** | **1.1E+11** | **6.0E+08** |
|  |  |  |  |  |  |
| Denmark | Starchy Roots | 535 | 9.9E+04 | 3.0E+08 | 1.1E+06 |
|  | Offals | 41 | 8.1E+05 | 6.4E+08 | 7.8E+05 |
|  | Vegetable Oils | 15 | 4.4E+04 | 1.2E+08 | 6.7E+05 |
|  | Pulses | 9 | 8.3E+03 | 2.4E+07 | 1.3E+05 |
|  | Meat | 711 | 7.9E+06 | 9.1E+09 | 5.0E+07 |
|  | Milk-Excluding Butter | 2137 | 3.0E+06 | 3.7E+09 | 1.7E+07 |
|  | Sugar&Sweeteners | 501 | 3.1E+05 | 4.8E+08 | 2.5E+06 |
|  | Alcoholic Beverages | 833 | 4.2E+05 | 5.3E+08 | 3.3E+06 |
|  | Eggs | 145 | 4.6E+05 | 4.7E+08 | 2.2E+06 |
|  | Animal Fats | 196 | 2.0E+06 | 1.0E+09 | 1.4E+07 |
|  | Cereals-Excluding Beer | 1072 | 1.5E+06 | 1.6E+09 | 8.6E+06 |
|  | Fish, Seafood | 226 | 8.6E+05 |  | 1.3E+06 |
|  | Miscellaneous |  |  |  |  |
|  | Oilcrops | 20 | 1.2E+04 | 7.5E+07 | 1.8E+05 |
|  | **Total** | **6442** | **1.7E+07** | **1.8E+10** | **1.0E+08** |
| Iceland | Starchy Roots | 417 | 7.7E+04 | 2.3E+08 | 8.3E+05 |
|  | Offals | 33 | 6.4E+05 | 5.0E+08 | 6.2E+05 |
|  | Vegetable Oils | 83 | 2.3E+05 | 6.5E+08 | 3.6E+06 |
|  | Pulses | 7 | 6.4E+03 | 1.9E+07 | 1.0E+05 |
|  | Meat | 810 | 9.0E+06 | 1.0E+10 | 5.7E+07 |
|  | Milk-Excluding Butter | 1879 | 2.6E+06 | 3.2E+09 | 1.5E+07 |
|  | Sugar&Sweeteners | 491 | 3.1E+05 | 4.8E+08 | 2.5E+06 |
|  | Alcoholic Beverages | 849 | 4.2E+05 | 5.4E+08 | 3.4E+06 |
|  | Eggs | 95 | 3.0E+05 | 3.1E+08 | 1.4E+06 |
|  | Animal Fats | 166 | 1.7E+06 | 8.8E+08 | 1.2E+07 |
|  | Cereals-Excluding Beer | 749 | 1.0E+06 | 1.1E+09 | 6.0E+06 |
|  | Fish, Seafood | 863 | 3.3E+06 |  | 4.8E+06 |
|  | Miscellaneous | 200 | 1.0E+04 | 3.7E+08 | 3.2E+06 |
|  | Oilcrops | 31 | 1.9E+04 | 1.1E+08 | 2.8E+05 |
|  | **Total** | **6674** | **2.0E+07** | **1.9E+10** | **1.1E+08** |
| Ireland | Starchy Roots | 632 | 1.2E+05 | 3.5E+08 | 1.3E+06 |
|  | Offals | 15 | 3.0E+05 | 2.3E+08 | 2.9E+05 |
|  | Vegetable Oils | 123 | 3.5E+05 | 9.6E+08 | 5.3E+06 |
|  | Pulses | 24 | 2.2E+04 | 6.5E+07 | 3.6E+05 |
|  | Meat | 721 | 8.0E+06 | 9.2E+09 | 5.1E+07 |
|  | Milk-Excluding Butter | 1846 | 2.5E+06 | 3.2E+09 | 1.5E+07 |
|  | Sugar&Sweeteners | 761 | 4.7E+05 | 7.4E+08 | 3.8E+06 |
|  | Alcoholic Beverages | 1307 | 6.5E+05 | 8.3E+08 | 5.2E+06 |
|  | Eggs | 80 | 2.5E+05 | 2.6E+08 | 1.2E+06 |
|  | Animal Fats | 116 | 1.2E+06 | 6.1E+08 | 8.6E+06 |
|  | Cereals-Excluding Beer | 1179 | 1.6E+06 | 1.7E+09 | 9.4E+06 |
|  | Fish, Seafood | 192 | 7.3E+05 |  | 1.1E+06 |
|  | Miscellaneous | 36 | 1.8E+03 | 6.5E+07 | 5.7E+05 |
|  | Oilcrops | 30 | 1.8E+04 | 1.1E+08 | 2.7E+05 |
|  | **Total** | **7062** | **1.6E+07** | **1.8E+10** | **1.0E+08** |
| Norway | Starchy Roots | 504 | 9.3E+04 | 2.8E+08 | 1.0E+06 |
|  | Offals | 17 | 3.3E+05 | 2.6E+08 | 3.2E+05 |
|  | Vegetable Oils | 34 | 9.5E+04 | 2.6E+08 | 1.5E+06 |
|  | Pulses | 50 | 4.6E+04 | 1.4E+08 | 7.5E+05 |
|  | Meat | 655 | 7.3E+06 | 8.4E+09 | 4.6E+07 |
|  | Milk-Excluding Butter | 1575 | 2.2E+06 | 2.7E+09 | 1.3E+07 |
|  | Sugar&Sweeteners | 415 | 2.6E+05 | 4.0E+08 | 2.1E+06 |
|  | Alcoholic Beverages | 679 | 3.4E+05 | 4.3E+08 | 2.7E+06 |
|  | Eggs | 114 | 3.6E+05 | 3.7E+08 | 1.7E+06 |
|  | Animal Fats | 111 | 1.2E+06 | 5.9E+08 | 8.2E+06 |
|  | Cereals-Excluding Beer | 1121 | 1.5E+06 | 1.6E+09 | 9.0E+06 |
|  | Fish, Seafood | 492 | 1.9E+06 |  | 2.8E+06 |
|  | Miscellaneous | 786 | 3.9E+04 | 1.4E+09 | 1.3E+07 |
|  | Oilcrops | 27 | 1.6E+04 | 9.9E+07 | 2.4E+05 |
|  | **Total** | **6579** | **1.6E+07** | **1.7E+10** | **1.0E+08** |
| Sweden | Starchy Roots | 517 | 9.6E+04 | 2.9E+08 | 1.0E+06 |
|  | Offals | 2 | 3.0E+04 | 2.4E+07 | 2.9E+04 |
|  | Vegetable Oils | 75 | 2.1E+05 | 5.8E+08 | 3.2E+06 |
|  | Pulses | 17 | 1.6E+04 | 4.6E+07 | 2.6E+05 |
|  | Meat | 727 | 8.1E+06 | 9.3E+09 | 5.1E+07 |
|  | Milk-Excluding Butter | 1727 | 2.4E+06 | 3.0E+09 | 1.4E+07 |
|  | Sugar&Sweeteners | 465 | 2.9E+05 | 4.5E+08 | 2.3E+06 |
|  | Alcoholic Beverages | 792 | 4.0E+05 | 5.1E+08 | 3.2E+06 |
|  | Eggs | 135 | 4.3E+05 | 4.4E+08 | 2.0E+06 |
|  | Animal Fats | 209 | 2.2E+06 | 1.1E+09 | 1.5E+07 |
|  | Cereals-Excluding Beer | 985 | 1.4E+06 | 1.4E+09 | 7.9E+06 |
|  | Fish, Seafood | 311 | 1.2E+06 |  | 1.7E+06 |
|  | Miscellaneous | 73 | 3.7E+03 | 1.3E+08 | 1.2E+06 |
|  | Oilcrops | 31 | 1.8E+04 | 1.1E+08 | 2.8E+05 |
|  | **Total** | **6067** | **1.7E+07** | **1.7E+10** | **1.0E+08** |
| United Kingdom | Starchy Roots | 888 | 1.6E+05 | 4.9E+08 | 1.8E+06 |
|  | Offals | 20 | 3.9E+05 | 3.1E+08 | 3.8E+05 |
|  | Vegetable Oils | 143 | 4.0E+05 | 1.1E+09 | 6.1E+06 |
|  | Pulses | 31 | 2.9E+04 | 8.5E+07 | 4.7E+05 |
|  | Meat | 859 | 9.5E+06 | 1.1E+10 | 6.0E+07 |
|  | Milk-Excluding Butter | 2115 | 2.9E+06 | 3.6E+09 | 1.7E+07 |
|  | Sugar&Sweeteners | 391 | 2.4E+05 | 3.8E+08 | 2.0E+06 |
|  | Alcoholic Beverages | 1043 | 5.2E+05 | 6.7E+08 | 4.2E+06 |
|  | Eggs | 119 | 3.8E+05 | 3.9E+08 | 1.8E+06 |
|  | Animal Fats | 50 | 5.1E+05 | 2.6E+08 | 3.7E+06 |
|  | Cereals-Excluding Beer | 1296 | 1.8E+06 | 1.9E+09 | 1.0E+07 |
|  | Fish, Seafood | 211 | 8.0E+05 |  | 1.2E+06 |
|  | Miscellaneous | 140 | 7.0E+03 | 2.6E+08 | 2.2E+06 |
|  | Oilcrops | 38 | 2.3E+04 | 1.4E+08 | 3.4E+05 |
|  | **Total** | **7.3E+03** | **1.8E+07** | **2.1E+10** | **1.1E+08** |
| **North European Countries** | Starchy Roots | 3494 | 6.5E+05 | 1.9E+09 | 7.0E+06 |
|  | Offals | 127 | 2.5E+06 | 2.0E+09 | 2.4E+06 |
|  | Vegetable Oils | 474 | 1.3E+06 | 3.7E+09 | 2.0E+07 |
|  | Pulses | 138 | 1.3E+05 | 3.7E+08 | 2.1E+06 |
|  | Meat | 4483 | 5.0E+07 | 5.7E+10 | 3.1E+08 |
|  | Milk-Excluding Butter | 11280 | 1.6E+07 | 1.9E+10 | 9.0E+07 |
|  | Sugar&Sweeteners | 3024 | 1.9E+06 | 2.9E+09 | 1.5E+07 |
|  | Alcoholic Beverages | 5504 | 2.8E+06 | 3.5E+09 | 2.2E+07 |
|  | Eggs | 687 | 2.2E+06 | 2.2E+09 | 1.0E+07 |
|  | Animal Fats | 847 | 8.8E+06 | 4.5E+09 | 6.3E+07 |
|  | Cereals-Excluding Beer | 6402 | 8.8E+06 | 9.3E+09 | 5.1E+07 |
|  | Fish, Seafood | 2295 | 8.8E+06 | 0.0E+00 | 1.3E+07 |
|  | Miscellaneous | 1235 | 6.2E+04 | 2.3E+09 | 2.0E+07 |
|  | Oilcrops | 177 | 1.1E+05 | 6.5E+08 | 1.6E+06 |
|  | **TOTAL** | **4.0E+04** | **1.0E+08** | **1.1E+11** | **6.3E+08** |

Legend: MFW: Metabolic Food Waste
